# Common Premotor Regions for the Perception and Production of Prosody and Correlations with Empathy and Prosodic Ability

**DOI:** 10.1371/journal.pone.0008759

**Published:** 2010-01-20

**Authors:** Lisa Aziz-Zadeh, Tong Sheng, Anahita Gheytanchi

**Affiliations:** 1 Brain and Creativity Institute, University of Southern California, Los Angeles, California, United States of America; 2 Department of Occupational Science, University of Southern California, Los Angeles, California, United States of America; 3 Neuroscience Graduate Program, University of Southern California, Los Angeles, California, United States of America; 4 Pacific Graduate School of Psychology, Palo Alto, California, United States of America; University of Groningen, Netherlands

## Abstract

**Background:**

Prosody, the melody and intonation of speech, involves the rhythm, rate, pitch and voice quality to relay linguistic and emotional information from one individual to another. A significant component of human social communication depends upon interpreting and responding to another person's prosodic tone as well as one's own ability to produce prosodic speech. However there has been little work on whether the perception and production of prosody share common neural processes, and if so, how these might correlate with individual differences in social ability.

**Methods:**

The aim of the present study was to determine the degree to which perception and production of prosody rely on shared neural systems. Using fMRI, neural activity during perception and production of a meaningless phrase in different prosodic intonations was measured. Regions of overlap for production and perception of prosody were found in premotor regions, in particular the left inferior frontal gyrus (IFG). Activity in these regions was further found to correlate with how high an individual scored on two different measures of affective empathy as well as a measure on prosodic production ability.

**Conclusions:**

These data indicate, for the first time, that areas that are important for prosody production may also be utilized for prosody perception, as well as other aspects of social communication and social understanding, such as aspects of empathy and prosodic ability.

## Introduction

Prosody, the melody and intonation of speech, involves the rhythm, rate, pitch and voice quality to relay linguistic and emotional information from one individual to another. A significant component of human social communication depends upon interpreting and responding to another person's prosodic tone as well as one's own ability to produce prosodic speech. However there has been little work on whether the perception and production of prosody share common neural processes, and if so, how these might correlate with individual differences in social ability.

The *production* of prosody is well known to be a specialization of the premotor cortex, in particular the inferior frontal gyrus (IFG), with emotional prosody more strongly activating the right hemisphere and linguistic prosody more strongly activating the left hemisphere [1], [2]. Research on the *perception* of prosody has largely focused on the right temporal lobe. However, despite this emphasis, there is some indication that the premotor cortex may also be involved [1], [3], [4]. Nevertheless, premotor contributions to prosody perception have not been well studied.

There is limited evidence that there may be common frontal areas active for both the perception and production of prosody; patients with lesions to frontal areas seem to have difficulty with both the perception and production of prosody [2]. However, these lesions are often very large and it is difficult to discern if the same brain areas are utilized in the two tasks. If the same areas were to be involved, it may indicate that, at least under some circumstances, the acoustic signals from another person's prosodic speech are transformed into articulatory signals in order to understand prosodic meaning. That is, it may imply that in order to understand someone else's prosodic intonation, we may utilize our own motor representations of how we would produce the given intonation.

Indeed, there is a growing body of data indicating that premotor areas are sensitive to the sounds of actions [5]–[7]. This activation is somatotopic, such that the sounds of hand actions activate the hand premotor areas and the sounds of mouth actions activate the mouth premotor areas [7]. The finding that regions in motor-related cortices are active for both the production and perception of a particular action is commonly referred to as “mirror system” activation. This data has also been extended for speech perception, showing that premotor mouth areas involved in producing speech are also involved in perceiving speech [8], [9]. The latter data indicate that motor areas may be involved in the processing of speech, particularly in noisy environments like the fMRI scanner room [10]. The current research investigates whether a similar pattern could be found for prosody. It also extends the findings of the auditory mirror system to include processing that is relevant to social and emotional information [11].

Furthermore, there is evidence that activity in premotor areas that respond to the sounds of actions correlates with one's ability to empathize with others [7]. This finding supports the idea that mapping the perception of other people's actions onto one's own motor representations (simulation) may be an important aspect of empathy. There is also evidence that individuals who score low on measures of empathy (as in psychopathic personality as well as autism) have poor prosodic ability [12]. Investigating the role of prosodic ability and its neural processes has clinical implications in clarifying the role of affective deficits in psychopathy. For this reason, we are particularly interested in exploring the relationship between prosody, empathy, and the mirror system.

## Materials and Methods

### Participants

Twenty right-handed, native-English speaking volunteers with no history of neurological or psychiatric conditions participated in the experiment. One subject was eliminated from all analyses due to technical errors, bringing the total to 19 subjects (13 females; 18–58 range, mean 28.1). All subjects had normal or corrected-to-normal vision and normal hearing. All assessments were made by screening questionnaires and all subjects gave informed written consent. Human subjects approval for this study was approved by the Institutional Review Board at the University of Southern California.

### Stimuli and Task Procedures

The main goal of the study is to determine if there are common regions for the production and perception of prosody. For this reason, the functional imaging component of the experiment consisted of two tasks, one to investigate prosody production and another to investigate prosody perception. Half of the subjects performed the production task runs first, while the other half performed the perception task first. Subjects were trained on the tasks prior to scanning.

#### Production task

**Figure 1:**
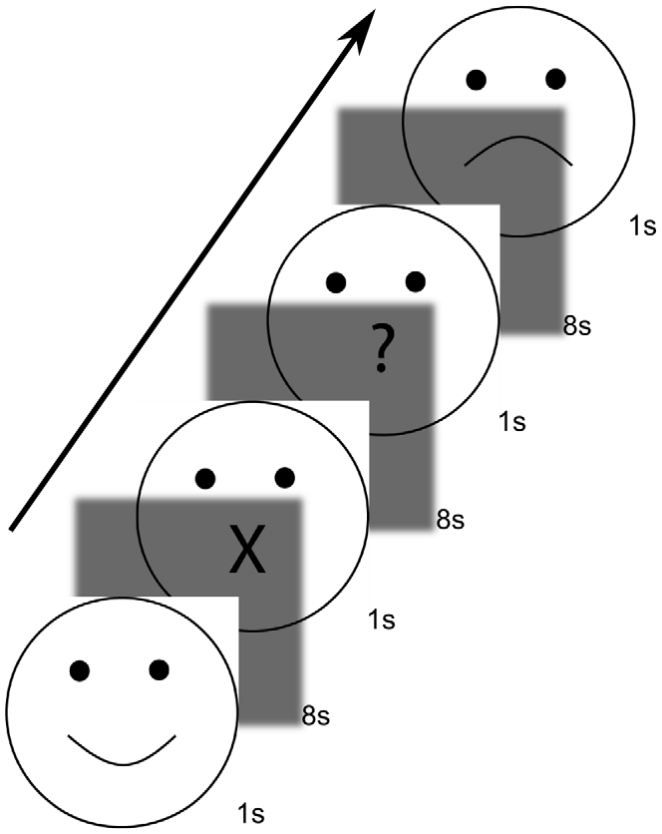
Schematic of the prosody production task design. A visual cue is presented 1 s, followed by 8 s of blank screen. Acquisition of functional volumes occurred during the last 2 s of the blank screen. The conditions were “happy”, “sad”, “question”, “neutral” (not shown in figure), and “rest”. The presentation order of the conditions was randomized for each subject.

Nonsense syllables were used to reduce/exclude additional linguistic processing (e.g., syntax, semantics) [13]. Subjects were asked to produce the phrase “da da da da da” in different intonations: happy, sad, question, and neutral. Participants were also instructed to produce no speech on some trials (rest condition). Note that our control condition, “neutral” intonation, will still contain intonation, as a flat pitch profile is still a pitch profile. However, it should nevertheless contain less prosodic information than the other conditions. Subjects were presented with a visual cue at the onset of each trial. A line drawing of a face was used to cue the participant to produce one of five task conditions (happy, sad, question, neutral, rest). As Figure 1 shows, the mouth of the line drawing varied for each cue (smile, frown, question mark for question, straight line for neutral, and X for rest). The visual cue was presented on the screen for 1 s followed by a gray screen and subjects were asked to produce speech as soon as the gray screen appeared. Subjects were trained prior to scanning to produce speech in a tone of voice that matched the presented visual cue. Each seven and a half minute functional run consisted of ten trials of each condition (including rest) for a total of 50 trials, and each subject performed three functional runs of the production task (30 trials per condition total). Participants' performance during the production task were monitored by an experimenter via headphones and recorded through an fMRI-safe microphone and digital voice recorder. Prior testing of the recording setup indicated that while the quality of the recordings were affected by the MRI background noise and conduction through the tubing, these degradations were minimal and did not affect subsequent analyses of voice data. A further concern when subjects produce speech is the possibility for motion artifacts. Our design minimized movement artifact by training subjects prior to scanning to move their heads minimally while producing speech, by using phrases that require minimal jaw movement (e.g.,“da”), and by using other sophisticated motion correction techniques (e.g., an on-line acquisition correction technique during scanning, and use of motion parameters as regressors in the analyses).

#### Perception task

The perception task had the identical design as the production task except for the stimuli; no visual stimuli were presented. Instead, each trial began with a delay of 1 s followed by an auditory stimulus of duration 2 s. The auditory stimuli consisted of voice recordings (“da-da-da-da-da” recorded by an actress) that depicted the conditions happy, sad, question, and neutral. As in the production task, nonsense syllables were chosen to minimize effects of semantics and syntax. Subjects were instructed to listen to the auditory stimulus and to especially attend to the intonation of the voice. All auditory stimuli were pre-tested prior to the experiment. As in the production task, each seven and a half minute functional run consisted of 10 trials of each condition, plus 10 trials where no auditory stimulus was delivered (rest trials), for a total of 50 trials. Each subject performed three functional runs of the perception task (30 trials per condition total).

### Image Acquisition

Functional MRI images were acquired with a Siemens MAGNETOM Trio 3T machine. In order to ensure that participants could hear the auditory stimuli during the perception task and that we could take audible voice samples during scanning of the production task, we used a sparse sampling paradigm throughout the experiment [14], [15]. In this paradigm, we minimized scanner noise by acquiring one volume 6 s after event onset to capture the peak of the hemodynamic response to the stimulus [16]. In the production task, volumes were acquired 6 s after the offset of the visual cue (which was approximately the onset of the subjects' speech production); in the perception task, functional acquisitions occurred 6 s following stimulus onset. Functional volumes were acquired with a echo planar T2*-weighted gradient echo sequence (TR = 9000 ms; TA = 2000 ms; TE = 30 ms; flip angle = 90°; 192 mm FoV; 64×64 voxel matrix; 29 axial slices (interleaved); 3×3×4.5 mm voxels, no gap). A high-resolution T1-weighted structural scan (MPRAGE; TR = 1950 ms; TE = 2.56 ms; flip angle = 90°; 256 mm FoV; 256×256 voxel matrix; 208 coronal slices; 1×1×1 mm voxels) as well as a T1-weighted structural scan with the same slice prescription as the functional images (coplanar; TR = 702 ms; TE = 17 ms; flip angle = 55°; FoV = 192 mm; 192×192 voxel matrix; 29 axial slices; 1×1×4.5 mm voxels) were also acquired from all subjects. Acquisition of functional volumes employed Siemens' prospective acquisition correction (PACE) technique for motion correction, in which head movements are calculated by comparing successively acquired volumes and are corrected on-line [17], [18].

### Image Processing

Functional images were preprocessed and analyzed with SPM2 software (www.fil.ion.ucl.ac.uk/spm/; Wellcome Department of Imaging Neuroscience, London, UK). Images were corrected for slice timing and then normalized to MNI space (using the EPI.mnc template) to allow across-subject comparisons. Motion parameters were calculated for the functional images. Images were then un-warped using the motion parameters and then spatially smoothed with a 7.5 mm Gaussian filter. In each task (production and perception), each condition (happy, sad, question, neutral, rest) was estimated with a Finite Impulse Response, and motion parameters were added to the design matrix as nuisance variables to minimize the effects of head movements during scanning. Scans were excluded from analysis if translational motion greater than 3 mm was detected; no participant exceeded this amount of translational motion. The finite impulse response model was used because our sparse sampling paradigm made it impossible for us to model the entire length/shape of the hemodynamic response function, and thus we needed to analyze each trial/volume as an impulse function. T-contrasts were computed to observe differences between conditions. Group analyses were performed using random effects models with contrast estimates from individual subjects and were thresholded at p<0.05 (FDR multiple comparisons correction) with a minimum cluster size of 5 contiguous voxels.

#### Task-related activity for prosody

To observe brain regions involved in the processing of prosody, we performed the contrasts “happy-neutral” and “question-neutral”. These contrasts were performed for the production and the perception task separately. The “happy-neutral” and contrast will reveal brain regions involved in emotional prosody processing, while the “question-neutral” contrast will reveal brain regions involved in linguistic prosody processing. The “sad” condition was not used in this analysis because 1) “happy” and “sad” emotions may be processed differently (e.g., Davidson's Approach-Withdrawal Hypothesis [19]); 2) if “sad” were included, then the “emotional” and “linguistic” prosody tasks will not be balanced; 3) acoustical analysis indicated that “sad” is more similar to the neutral prosody condition than the “happy” condition, and different from both “happy” and “question” conditions (see supplementary materials, File S1). Thus omitting the “sad” condition from this analysis allows us to maximize the difference between our control condition and question prosody condition.

#### Common regions for perception and production of prosody

To determine brain regions involved in both the production and the perception of emotional prosody, we observed whether regions associated with emotional prosody production were also active during emotional prosody perception. The same procedure was applied for linguistic prosody production and perception. We first obtained a thresholded map for the production task contrast (“happy-neutral” for emotional prosody; “question-neutral” for linguistic prosody; p<0.05, FDR, k>5). Individual clusters from the thresholded production contrast maps were then used as masks to determine whether prosody perception also activated voxels within those regions. These masks were then used to apply small volume correction (SVC) to the corresponding prosody perception contrasts.

### Behavioral Measures

We were further interested in how activity in brain areas involved in prosody production/perception may correlate with an individual's ability to produce or perceive prosody. Furthermore, because of the relationship between prosody perception and empathy described in clinical literature [12], we were also interested in finding a correlation between brain regions active during prosody perception and an individual's scores on measures of affective empathy. Thus, in addition to the fMRI experiment, we also administered questionnaires to our participants outside of the scanner in order to obtain measures of prosody ability and empathy. These measures were used to correlate prosodic ability to empathy as well as with the functional activations during the fMRI experiment.

#### Assessment of prosodic ability

To assess prosody *production* ability, two raters subjectively scored the voice recordings taken from participants during the fMRI production task on the level of expression of a subset of the trials. The scoring was performed after the scanning session. A 5-point Likert scale was used to judge prosodic ability, with “1” corresponding with “could not determine intended condition”, to “5” corresponding with “could absolutely determine intended condition; superb expression.” Three randomly selected “happy” and “sad” trials from each scanning run were scored, and average scores for “happy”, “sad”, and “happy&sad” were obtained for each subject. To assess prosody *perception* ability, we administered a separate questionnaire where subjects listened to 28 audio clips depicting the conditions happy, sad, question, and neutral, and were to determine the four conditions each clip belonged to. An accuracy score of the proportion of correctly determined clips was obtained for each subject as a measure of how well a person can distinguish between different prosody conditions.

#### Assessment of empathy

To obtain a measure of empathy in our subjects, we administered two questionnaires: the Interpersonal Reactivity Index (IRI) [20] and the Psychopathic Personality Inventory-Revised (PPI-R) [21]. The IRI, a self-report measure assessing specific dimensions of empathy, consists of 4 subscales, each measuring a unique component of empathy. As our aim was to correlate emotional aspects of empathy with individual ability to perceive emotional prosody, we focused on the component of the IRI thought to reflect an affective component of empathy, Personal Distress (PD; e.g., “When I see someone who badly needs help in an emergency, I go to pieces” [20]. The other subscales of the IRI are Fantasy Scale (FS), Empathic Concern (EC), and Perspective Taking (PT). EC is another form of affective empathy, while FS and PT are considered to be cognitive forms of empathy. These subscales were not included in the hypotheses. The PPI-R also consists of multiple subscales and factors, each representing some psychopathic personality trait. The affective component of psychopathic personality has generally been thought to be inversely related to empathy; individuals who exhibit psychopathic personality traits and show symptoms of antisocial personality disorder are also likely to show callousness and a lack of empathy [22], [23]. Specifically, the Coldheartedness scale (C) of the PPI-R reflects a propensity toward callousness, guiltlessness, and lack of sentimentality, and is related to a lack of affective empathy. Thus, the PPI-R Coldheartedness scale was used as an additional measure of affective empathy, and we predicted that it would negatively correlate with prosody perception.

### Correlations between Prosody Perception and Empathy

#### Behavioral

To determine whether an individual's ability to perceive prosody is related to their empathy, we performed correlations between subjects' scores on the prosody perception questionnaire and empathy scores. Once again we focused on components of empathy and performed correlation analyses using subscales that relate specifically to affective empathy, the Personal Distress scale of the IRI and the Coldheartedness scale of the PPI-R.

#### fMRI

To determine prosody-related brain regions whose activity correlates with prosody perception and empathy ability, we ran simple regression models at the group level for the contrast “happy&sad-neutral” using individuals' empathy scores as regressors. To observe which brain regions show a linear relationship to empathy, contrast estimates of “happy&sad-neutral” perception were correlated with PD scores from the IRI and C scores from the PPI-R to elucidate correlations between affective empathy and neural activity during emotional prosody perception. These analyses were thresholded at p<0.005 uncorrected with a cluster threshold of k>5 voxels. Both the “happy” and “sad” conditions were included in this analysis as we posited that the neural systems involved in perceive both these intonations was related to empathic ability.

### Prosody Production Ability Correlated with Emotional Prosody Production

Do individuals who are better at producing prosody show more activity in motor regions involved in prosody production? To investigate this we correlated areas that were active for emotional prosody production with the behavioral measure of prosody production ability. To observe which brain regions show a linear relationship to prosody production ability (i.e., the voice production ratings), we correlated each subject's “happy&sad-neutral” production task contrast estimates with their voice ratings.

## Results

### Task-Related Activity for Prosody

#### Emotional prosody production

The contrast “happy-neutral” for the production task revealed activations in the left inferior frontal gyrus, bilateral anterior middle temporal gyri, bilateral lingual gyri, left cuneus, right midbrain, right fusiform gyrus, left middle frontal gyrus, right anterior cingulate gyrus, bilateral thalami, left superior frontal gyrus, right middle occipital gyrus, left middle cingulate gyrus, right caudate, right insula, left anterior superior medial gyrus, and bilateral posterior superior medial gyri (p<0.05, FDR, k>5). A complete list of results is available in the supplementary materials (File S1). In addition, a whole-brain contrast against rest is shown in Figure S3 and against control in Figure S4. Regions specifically involved in emotional prosody perception as compared to control are shown in Figure S5.

#### Linguistic prosody production

The contrast “question-neutral” for the production task revealed widespread activations across many regions, including portions of the superior, middle, and inferior frontal gyri bilaterally, the supplementary motor area, medial regions of the parietal and occipital cortices, the lingual gyri bilaterally, portions of the left insula, posterior regions of the middle temporal gyri bilaterally, the left superior temporal gyrus, and portions of the anterior cingulate cortex (p<0.05, FDR, k>5. A complete list of results is available in the supplementary materials (File S1). In addition, a whole-brain contrast against rest is shown in Figure S3 and against control in Figure S4. Regions specifically involved in linguistic prosody perception as compared to control are shown in Figure S5.

### Shared Networks for Emotional Prosody

**Figure 2:**
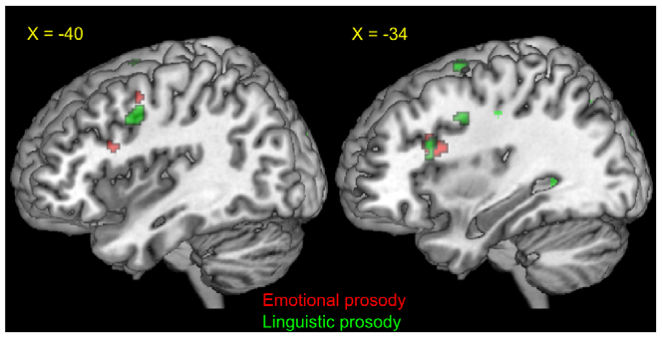
Regions of overlap between prosody production and perception. Red = Emotional prosody production regions (p<0.05, FDR; T>3.48) that were also active for perception (p<0.05, FDR (SVC); T>2.38). Green = Linguistic prosody production regions (p<0.05, FDR; T>3.80) that were also active for perception (p<0.05, FDR (SVC); T>2.45). A region in the left inferior frontal gyrus appears to be involved for the production and perception of both emotional and linguistic prosody.

In order to determine whether brain regions active while producing emotional prosody were also active when perceiving emotional prosody, we created masks from the thresholded production contrast “happy-neutral”, and observed whether these regions were also active in perception. Masks from the production contrast were used to perform small volume corrections to the perception contrast “happy-neutral”. As predicted, motor related regions in the left inferior frontal gyrus (pars opercularis; BA44) and the left middle frontal gyrus (BA 6; dorsal premotor cortex) were significantly active. The left middle cingulate gyrus, right caudate, and right thalamus also survived SVC (p<0.05, FWE, k>5) (Figure 2).

### Shared Networks for Linguistic Prosody

We further predicted that motor-related regions would be commonly active for the perception and production of linguistic prosody. In support of our hypothesis, motor-related regions including the left inferior frontal gyrus (pars opercularis; BA44) and left middle frontal gyrus (BA 6; dorsal premotor cortex) and bilateral superior frontal gyri (BA 6) were active for both tasks. The left anterior cingulate cortex and left insula also survived SVC (p<0.05, FWE, k>5) (Figure 2).

### Behavioral Results

#### Empathy scales

*IRI*. All 19 subjects completed the IRI. The mean scores (and standard deviations) for each subscale are as follows: FS = 19.21 (5); EC = 18.63 (4.7); PD = 8.58 (5.31); PT = 18.26 (5.94). These values are similar to those originally reported by Davis (1980) and are within two standard deviations of the normed mean. *PPI-R*. One subject did not complete the PPI-R due to experimental difficulties; one participant reported 2 standard deviations above the mean and the remaining 17 participants scored within normal range (+/− 1.5 SD) (mean = 29.16; std = 6.33). As the PPI was originally normed on a college population, our patterns reflect the normal bell curve expected for this measure.

#### Correlations between prosody perception ability and empathy

As expected, correlations between behavioral measures of prosody and empathy revealed significant results for the PD scale of the IRI and for the C scale of the PPI-R. The PD scale correlated positively with performance on the prosody perception task (r = 0.46; R-sq = 0.21; p(one-tailed) <0.0287). This finding is consistent with our prediction that prosody perception ability will be related with affective empathy. The C scale was found to correlate negatively with performance on the prosody perception task (r = −0.47; R-sq = 0.22; p(one-tailed) <0.0297). Because the C scale is an indicator of deficits in affective empathy, the finding of a negative correlation between C scale scores with prosody perception is expected. It should be noted that in future studies, larger sample sizes would be more optimal in testing these scales, and further allow for more stringent analyses to test the hypotheses. Graphs of performance and production scores are shown in Figure S1 and scatter plots for these correlations are shown in Figure S2.

### Affective Empathy Scores Correlated with Emotional Prosody Perception: fMRI Results

**Figure 3:**
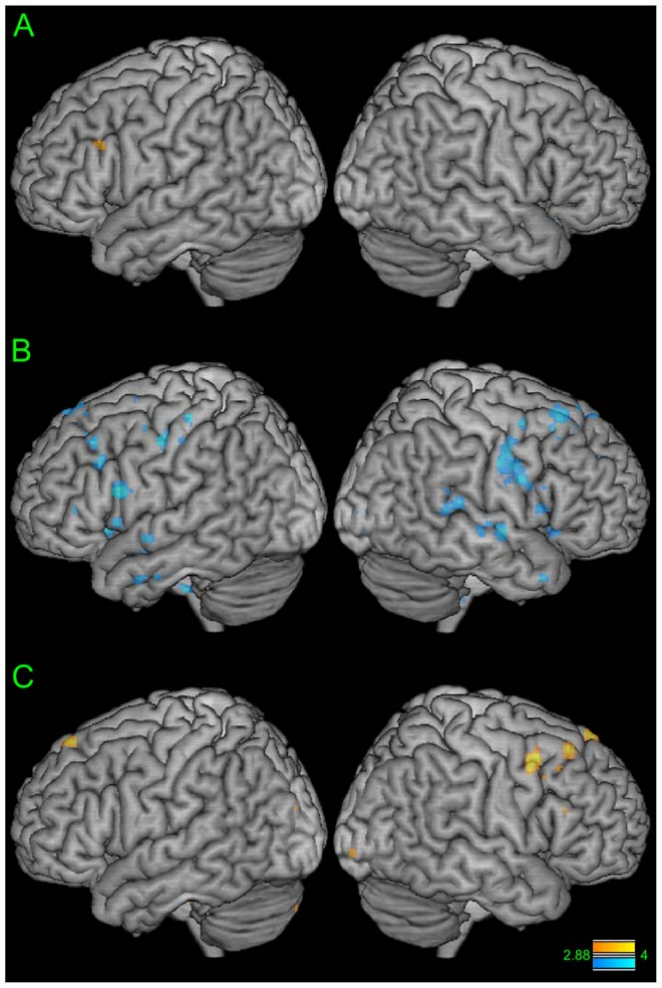
Regions involved in emotional prosody perception correlated with empathy. **A**) Correlation between emotional prosody perception brain regions and individual differences in PD (IRI) scores. Orange  =  regions that show positive correlation (p<0.005 uncorrected; Z>2.88). **B**) Correlation between emotional prosody perception brain regions and individual differences in C (PPI-R) scores. Blue  =  regions that show negative correlation (p<0.005 uncorrected; Z>2.88). **C**) Correlations between emotional prosody production brain regions and performance on prosody production task (rating scores) (p<0.005 uncorrected; Z>2.88).

A correlation analysis between individual differences in the PD score from the IRI and contrast estimates during emotional prosody perception indicates regions in the left inferior frontal gyrus (pars triangularis) and right cerebellum as showing activity that positively correlates with PD scores (p<0.005; uncorrected, k>5) (Figure 3a). The R-sq for the left IFG is 0.42 with a 95% confidence interval of 0.12–0.73.

For the PPI-R, higher scores in the cold-heartedness scale (C) indicate deficits in empathic ability. Thus here we focused on a negative correlation with the C score and neural activity during emotional prosody perception. A correlation analysis between individual differences in the C score from the PPI-R and contrast estimates during emotional prosody perception indicates regions in the frontal cortex, including bilateral superior, middle, and inferior frontal gyri, bilateral cingulate sulcus, bilateral anterior insula, bilateral transverse temporal gyrus (Heschl's gyrus), bilateral superior temporal gyrus, and right TPJ show activity that negatively correlates with C score (p<0.005; uncorrected, k>5) (Figure 3b). The R-sq for a region within the left inferior frontal gyrus (pars opercularis) is 0.54 with a 95% confidence interval of 0.26–0.92. While the results reported here support our hypotheses, it should be noted that larger sample sizes would greatly reinforce this finding, and would better allow for effects to be tested with more stringent tests.

Further post-hoc analyses in the perception/IRI and perception/PPI-R analyses, indicate that the correlations are driven in part by processing of neutral stimuli. Whereas we report a positive correlation in the left inferior frontal sulcus between PD score (IRI) and the “happy&sad - neutral” contrast, this correlation is influenced by a negative correlation between activity during “neutral” and PD score. Likewise, in the left inferior frontal gyrus (L IFG), we report a strong negative correlation between the Coldheartedness score (C; PPI-R) and the “happy&sad - neutral” contrast. This correlation is also in part influenced by a positive correlation between C score and “neutral” activity.

### Prosody Production Ability Correlated with Emotional Prosody Production

A linear regression between individual differences in prosody production ability and contrast estimates during emotional prosody production indicates motor-related regions in the right inferior frontal gyrus (pars triangularis), the left superior frontal gyrus, and right middle frontal gyrus to be positively correlated to prosody production ability (p<0.005; uncorrected, Figure 3c), although this result did not meet the cluster threshold of k>5 voxels. The R-squared for this result is 0.36 with a 95% confidence interval of 0.04–0.67.

## Discussion

### Common Brain Regions for the Production and Perception of Prosody

We found areas in the premotor cortex, including the left inferior frontal gyrus and the left dorsal premotor cortex were active for both the perception and production of prosody. This was true for both emotional prosody and linguistic prosody. These results are consistent with previous findings of activity in premotor regions during prosody perception [1], [24]. The current result indicates a link between perception and production, where brain areas that are commonly thought to be involved with motor planning are also active for perception. While there have been numerous previous reports of perceptual processing in motor areas for action observation [25]–[27], for the sounds of actions [6], [7], and even for speech [8], to our knowledge this is the first report of “mirror” processing for prosody. It may indicate that some components of prosodic perception involve mapping the heard speech to areas that are important for producing that same speech. Such mapping of acoustic signals to articulatory signals is reminiscent of the motor theory of speech perception [28]. This finding is also in line with the proposed “‘as-if’ body loop” where individuals utilize sensory-motor regions to implicitly simulate perceived or imagined experiences [29], as well as other studies that indicate that frontal regions are involved in prosodic perception [1], [3], [30], [31]. While we do not state that this is the only way that prosodic perceptual processing occurs (and clearly other regions are found to be active when just comparing prosody perception to control), activity in the premotor regions might contribute to the processing more or less strongly in particular circumstances, such as in subtle or more ambiguous instances [10]. Indeed, the topic of motor contributions to speech processing has been a subject of great debate [32], [33], and we take the view that motor contributions to speech processing are one several processing strategies that may be utilized, depending on speech context (e.g., noisy/quiet) [10] and the task demands.

The inferior frontal gyrus and premotor cortices are known to have connections to auditory areas, in particular though the arcuate fasciculus [5]. This “dorsal stream” of speech perception from auditory regions to inferior frontal regions may provide a sensory-motor interface that is important for mapping perceived speech onto articulatory processes [34], [35]. Thus, inferior frontal areas have the possibility for auditory and motor processing, and in fact are known to respond to the sounds of a variety of hand and mouth actions [7]. In the case of prosody, we hear our own prosody as we produce it. With time, co-activation of production and perception, through Hebbian learning, could strengthen the activity in multimodal premotor areas to either the afferent or efferent component of the speech, thus producing the areas that we find in this study to be active for both perception and production of prosodic speech.

Interestingly, our data indicate that common motor areas for production and perception of prosody were found in only the left hemisphere (left IFG and premotor cortices). This was true for both linguistic and emotional prosody. Thus, while emotional prosody perception and also prosody production are known to activate the right hemisphere each [2], “mirror” regions for prosody seem to be stronger in the left hemisphere. This is consistent with all previous reports of an auditory mirror system as being lateralized to the left hemisphere [6], [7], and may indicate a special role in the left premotor cortex for more multimodal processing (motor, visual, and auditory), while the right equivalent areas instead may be stronger in motor and visual properties rather than auditory properties.

One possible limitation in this analysis is the possibility that participants implicitly made facial movements during perception trials. Outside the scanner, electromyographic recordings were taken from some subjects to test this possibility, and these results of this analysis, indicating a lack of facial muscle movement during perception trials, are included in the supplementary materials (File S1). However it should be noted that any study on perception is limited by the possibility of implicit movement unless measured directly inside the scanning session.

### Correlations with Affective Empathy

Prosodic ability is known to correlate with deficits associated with affective components of empathic processing. This is best observed in individuals with psychopathy. These individuals, who often score low on emotional aspects of empathy, also tend to score poorly on the ability to perceive prosody [12]. Our behavioral results further support a positive correlation between ability to perceive prosody and ability to feel emotional aspects of empathy, constructs measured by the PPI-R scale of cold-heartedness (C) and the IRI scale of personal distress (PD). Thus we also looked at individual differences in emotional components of empathy [lower scores on (C) measure on the PPI-R, and personal distress (PD) measure on the IRI], and correlated these with areas that were active for the perception of emotional prosody. We found that individuals who scored higher on these measures of empathy showed more activity during emotional prosody perception in anatomically the same premotor areas that we previously found to be active for the perception and production of prosody, including the bilateral inferior frontal gyrus and premotor cortex. They also were found to show less activity in this region during neutral prosodic intonation, indicating that more empathic individuals utilize premotor regions for emotional prosodic perception, but less for non-emotional stimuli. This data support the notion that components of empathy to emotional stimuli may rely on simulation processes carried out, in part, by motor-related areas [7], [36]. Thus, in order to understand someone else's prosodic intonation, we may simulate how we would produce the given intonation ourselves, which in turn may be a component of the process involved in creating empathic feeling for that individual. These data indicate that individuals who score higher on scales of affective empathy also show more activity in motor-related areas during prosody perception. Our findings extend previous correlations between the mirror neuron system and individual differences in empathy to include, for the first time, an emotional auditory stimulus: happy or sad prosodic intonation.

The negative correlation with the C score showed additional areas in the left anterior insula and the superior temporal gyrus. The insula activation might indicate more emotional processing when perceiving emotional stimuli by individuals who are more empathic. Activity in temporal areas may indicate that individuals who are more empathic might also initially process the perceived intonation more than other individuals as well. It is interesting to note that the motor-related activations are bilateral while the temporal activations are observed only in the right hemisphere. The right hemisphere temporal activations are consistent with previous studies of prosody perception; however the motor activities are instead consistent with the bilateral control of the mouth muscles, important for prosody production (see supplementary materials, File S1).

### Correlations with Prosodic Ability

Correlations between behavioral measures of prosody production ability and brain regions that are active during prosody production indicate that individuals who are better at producing prosody activate areas important for motor planning of prosody more than individuals that are poor at prosody production. Because here we focus on affective prosody production alone, we find activity predominately in the right hemisphere, as one would expect. While such a finding has been found for other areas of motor expertise [37], this is the first time we find such an effect for aspects of non-verbal aspects of language processing. A similar correlation for prosody perception, while interesting, was not possible due to a ceiling effect on the behavioral measures of perception ability; an abnormal population may be more relevant for such a correlation.

## Supporting Information

File S1 Supplementary materials text.
(0.20 MB DOC)

Figure S1 Prosody perception and production performance. Participants performed the prosody perception task with high accuracy, with a mean accuracy score of 0.91 (SD 0.11). The mean rating on the production task was 2.97 (SD 0.70).
(1.30 MB TIF)

Figure S2 Correlations between prosody perception performance and empathy measures. Participants' performance on the prosody perception task was positively correlated with PD scores, a measure of affective empathy [r = 0.46; R-sq = 0.21; p(one-tailed) <0.0287]. Prosody perception performance was negatively correlated with C scores, a measure thought to be associated with a lack of empathy [r = −0.47; R-sq = 0.22; p(one-tailed) <0.0297].
(0.30 MB TIF)

Figure S3 Regions involved in speech production and perception. Regions involved in the production (red; p<0.05 FDR, T>2.14) and perception (green; p<0.05 FDR, T>2.83) of all speech conditions (all speech - rest). Regions common to both production and perception are shown in yellow.
(1.51 MB TIF)

Figure S4 Figure S4: Regions involved in prosody production. Regions involved in the production of “happy” (red; p<0.05; FDR; T>3.48), “sad” (blue; p<0.05; FDR; T>4.75), and “question” (green; p<0.05; FDR; T>3.80) prosody (compared against the “neutral” condition.
(5.07 MB TIF)

Figure S5 Regions involved in prosody perception. Regions involved in the perception of “happy” (red), “sad” (blue), and “question” (green) prosody (compared against the “neutral” condition). All effect size maps were thresholded at p<0.005 (uncorrected). No voxels in any of the three contrasts survive multiple comparisons correction at the whole-brain level.
(5.06 MB TIF)
