# Supplementary material for: Common Premotor Regions for the Perception and Production of Prosody and Correlations with Empathy and Prosodic Ability
Source: PLoS One. 2010 Jan 20;5(1):e8759. doi: 10.1371/journal.pone.0008759 (PMC2808341; doi:10.1371/journal.pone.0008759)
Supplement: File S1 — Supplementary materials text. (0.20 MB DOC) [file pone.0008759.s001.doc]

**Supplementary Materials**

**Stimuli Pretesting Methodology**

Twelve subjects participated in pre-testing of stimuli. In a quiet room, subjects listened to 30 different stimuli from each condition (happy, sad, neutral, question). After each condition, subjects were asked to categorize the stimulus and rate how well they were able to tell the categorization. One stimulus for each category was chosen based on this pretesting, and for each category, it was the stimulus that all subjects categorized correctly and reported ease with categorization.

**Additional Results and Discussion:**

*Behavioral results*

Prosody Production Ability. One subject’s voice recording data during the production task was lost due to a technical error. Thus data from 18 subjects were rated by two unbiased judges. The judges were trained to give ratings based on the defined criteria and strong inter-rater reliability was observed (r = 0.89, p < 0.05). In general subjects performed the prosody production task at an acceptable level (mean rating score = 2.97 out of 5; std = 0.70). These results are depicted in Figure S1.

Prosody Perception Ability. One subject was unable to complete the prosody perception task after the scanning session (different subject from above). The 18 subjects who did complete the perception task performed well (mean accuracy = 0.91; std = 0.11). These results are depicted in Figure S1. The level of performance and small observed variance are not surprising because conventional measures of prosody perception ability are typically not sensitive unless used with special subject groups, such as children with autism. As such, normal individuals are known to perform superiorly in prosody tasks (Peppe, Maxim J, & Wells B, 2000).

Prosody Production Acoustic Analyses. We compared voice recordings of the participants across the three pitch attributes: fundamental frequency, intensity, and duration. Voice pitch features were extracted and analyzed for every trial, for each subject, using Praat software ([http://www.praat.org](http://www.praat.org/)). We found that “happy” and “sad” voice recordings differed in fundamental frequency (P<0.025) and intensity (p<0.040), and that “happy” and “neutral” differed in fundamental frequency (p<0.016). No other acoustic differences in pitch were observed. Summary statistics of the pitch features are shown in Table S1.

*fMRI*

*Task-related activity for all speech* To observe brain regions involved during general speech processing, we performed the contrast “all-rest” for the production task and the perception task separately, where conditions happy, sad, question, and neutral were contrasted against the rest condition. These contrasts reveal brain regions that are involved during the task that are related to speech production and perception in general.

Speech Production-rest*.* The contrast “all-rest” for the *production* task revealed robust (p<0.05, FDR; k>5), widespread activations in right ventral precentral gyrus, bilateral superior temporal gyri, right inferior temporal gyrus, bilateral posterior orbital gyri, right superior parietal gyrus, and right cerebellum.The activation map overlaid onto the cortical surface is shown in Figure S3. Complete results shown in Table S2.

Speech Perception-Rest. The contrast “all-rest” for the *perception* task revealed activation patterns that essentially mirrors that of the production task (p<0.05, FDR; k>5). Activations were observed in much of the same premotor (including inferior frontal) and superior temporal regions as the production contrast (Figure S3). A complete table of the activation clusters is shown in Table S3.

*Discussion on Visual Cues*

Due to sparse sampling, we cannot dissociate the speech production task from the visual cue. While we do not believe that our regions (ventral premotor cortices) are active in response to the line drawings of faces, we have attempted to look at this issue in another experiment. Four different participants completed a speech production fMRI task similar to the task currently described. Continuous acquisition was used instead of sparse sampling in this experiment. A visual cue appeared for 1s, instructing the subjects to produce speech (da’s) according to an emotional condition. After the visual cue disappeared from the screen, a fixation is presented for 1.25-4.75 seconds. After that jittered interval, a go signal appeared, indicating to the subject to produce speech according to the visual cue. This design allowed for the separation of the visual and speech components. Preliminary results indicate that in fact the production task alone (factoring out the cued stimulus) shows robust activation in the inferior frontal gyrus (p<.001, uncorrected). These preliminary results support our interpretation that the activations observed in our production task are primarily related to the speech production task and not the visual cue.

*Other Effects:*

As a post-hoc analysis, we considered how age and order effects might have influenced our data. Because this was a post-hoc analysis for which we had no direct hypotheses, we used a threshold of p<.05, corrected for multiple comparisons (FDR correction). We did not find any evidence for order or age effects. Age effects are described in more detail below.

Age. We have recruited a broad sample for this study, and as such, we observe a large range in the age of our participants (mean = 28.1; median = 26; std = 10.7). Because an important component of our study involves correlation analyses, we hereby consider the possible influence of age as a predictor variable. From inspecting the correlation matrix and the R-squared matrix, we can conclude that Age is not a strong predictor of any other variable of interest, and thus was not studied further (Table S4).

Electromyography Recordings. Neuroimaging and electrophysiology studies involving the mirror neuron system are sometimes accompanied by control experiments using electromyography recordings from target muscles in order to rule out interpretations involving muscle activation (Ferrari, Gallese, Rizzolatti, & Fogassi, 2003; Gallese, Fadiga, Fogassi, & Rizzolatti, 1996). This is especially relevant to our experiment as we would like to ensure that the premotor activations we observe in the perception task were not in fact due to subvocal motor activity. While we did not perform simultaneous EMG recording while participants performed the fMRI task, we recorded EMG signals from a different group of participants outside of the scanner.
 Five naïve subjects (3 female) performed a variation of the task while EMG signals were recorded from the hyoglossus (tongue retractor muscle). The hyoglossus was chosen from among three candidate sites (masseter, larynx) because it had the most reliable signal as well as the best signal to noise ratio as indicated from pilot testing. EMG signals were recorded using Ag-AgCl cup electrodes (8mm) which maintained skin contact with application of electrode gel. Signals were recorded and filtered (gain = 5000, sample rate 500Hz) using the Biopac MP150 starter system (Biopac Systems, Inc; [http://www.biopac.com](http://www.biopac.com/)).

Subjects were instructed to either produce speech or remain silent depending on the visual cue. Short audio clips similar to the fMRI task were intermixed among the production and silent trials, and subjects were simply told to listen to the clips (i.e., perception trials). Recorded EMG signals were separated by condition and concatenated across subjects within a condition. The concatenated EMG signal is then root-mean squared and a sparse sample (effective reduction to ~3.3% of original sample rate) of this data was selected for the analyses to avoid biases towards significant results.
 Paired T-tests between concatenated EMG signals recorded during Production and Perception trials showed that the EMG activity from the hyoglossus was significantly different between task conditions (p=0.027, T=2.32). Moreover, the difference between Perception and Silent trials was not significant(p=0.977, T=0.03). Although a failure to reject the null hypothesis in this comparison is not sufficient to claim that there is no difference between Perception and Silent trials, the data strongly suggest the absence of subvocal motor activity during Perception trials.

**References for supplemental materials**

Calvo-Merino B, Glaser DE, Grezes J, Passingham RE, Haggard P (2005) Action observation and acquired motor skills: an FMRI study with expert dancers. Cereb Cortex 15:1243-1249.

Ferrari, P. F., Gallese, V., Rizzolatti, G., & Fogassi, L. (2003). Mirror neurons responding to the observation of ingestive and communicative mouth actions in the monkey ventral premotor cortex. *European Journal of Neuroscience*, *17*(8), 1703-1714.

Gallese, V., Fadiga, L., Fogassi, L., & Rizzolatti, G. (1996). Action recognition in the premotor cortex. *Brain: A Journal of Neurology*, *119 ( Pt 2)*, 593-609.

Hickok, G. (2004). Functional Anatomy of Speech Perception and Speech Production: Psycholinguistic Implications. *Journal of Psycholinguistic Research*, 30(3).

Hugdahl, K., Thomsen, T., Ersland, L., Morten Rimol, L., & Niemi, J. (2003). The effects of attention on speech perception: An fMRI study. *Brain and Language*, *85*(1), 37-48.

Scott, S. K., & Wise, R. J. S. (2003). PET and fMRI studies of the neural basis of speech perception. *Speech Communication*, *41*(1), 23-34.

Sekiyama, K., Kanno, I., Miura, S., & Sugita, Y. (2003). Auditory-visual speech perception examined by fMRI and PET. *Neuroscience Research*, *47*(3), 277-287.

Skipper, J. I., Nusbaum, H. C., & Small, S. L. (2005). Listening to talking faces: motor cortical activation during speech perception. *NeuroImage*, *25*(1), 76-89.

Soros, P., Sokoloff, L. G., Bose, A., McIntosh, A. R., Graham, S. J., & Stuss, D. T. (2006). Clustered functional MRI of overt speech production. *NeuroImage*, 32(1), 376-387.

**Table S1: Acoustic pitch parameters of prosody production.**

|  | Condition | Mean | Standard deviation | P < 0.05 |
| --- | --- | --- | --- | --- |
| F0 (hz) | happy | 197.36 | 52 | x+ |
|  | sad | 159.55 | 44.15 | x |
|  | neutral | 157.28 | 42.37 | + |
|  | question | 166.12 | 45.42 |  |
|  |  |  |  |  |
| Intensity (dB) | happy | 75.47 | 3.91 | x |
|  | sad | 73.08 | 2.63 | x |
|  | neutral | 73.68 | 3.22 |  |
|  | question | 74.75 | 4.22 |  |
|  |  |  |  |  |
| Duration (s) | happy | 2.06 | 0.76 |  |
|  | sad | 2.32 | 0.9 |  |
|  | neutral | 2.23 | 0.83 |  |
|  | question | 2.38 | 1.13 |  |

Acoustic pitch parameters from prosody production voice recordings. Measurement units for fundamental frequency (F0) was in hz, intensity in decibels (dB), and duration in seconds. Conditions “happy” and “sad” differed in fundamental frequency, as did “happy” and “neutral” (p<0.05 for both). Conditions “happy” and “sad” differed in mean intensity (p<0.05). No other differences were observed.

**Table S2: All – Rest prosody production contrast results.**

| **Cluster size (k)** | **p(FDR-cor)** | **Voxel T** | **x,y,z {mm}** | **Anatomical region** |
| --- | --- | --- | --- | --- |
| 87603 | 0.000 | 15.480 | 52 -6 16 | R Rolandic Operculum |
|  | 0.000 | 12.780 | 60 -28 4 | R Superior Temporal Gyrus |
|  | 0.000 | 11.390 | -58 -22 4 | L Superior Temporal Gyrus |
| 14 | 0.022 | 2.630 | 64 -54 -10 | R Inferior Temporal Gyrus |
| 35 | 0.025 | 2.560 | -2 -20 30 | L Posterior Cingulate Gyrus |
| 14 | 0.028 | 2.490 | -22 48 -8 | L Superior Orbital Gyrus |
| 7 | 0.033 | 2.390 | 20 54 -10 | R Superior Orbital Gyrus |
| 5 | 0.040 | 2.280 | 20 -62 52 | R Superior Parietal Lobule |
| 6 | 0.040 | 2.280 | 10 -74 -42 | R Cerebellum |

Height threshold: T=2.14, p=0.023

Extent threshold: k=5 voxels, p=0.87

Expected false discovery rate: <=0.05

df: 1, 18

Smoothness (FWHM): 13.5 13.9 13.3mm = 6.8 7.0 6.6voxels

Search volume: 1516016cmm, 189502voxels, 563.9resels

Voxel size: 2 2 2mm (1 resel=311.35voxels)

**Table S3: All – Rest prosody perception contrast results.**

| **Cluster size (k)** | **p(FDR-cor)** | **Voxel T** | **x,y,z {mm}** | **Anatomical region** |
| --- | --- | --- | --- | --- |
| 9614 | 0.000 | 9.99 | 60 -18 4 | R Superior Temporal Gyrus |
|  | 0.000 | 8.66 | 60 -32 10 | R Superior Temporal Gyrus |
|  | 0.000 | 8.65 | 56 14 16 | R Inferior Frontal Gyrus (p. opercularis) |
| 7946 | 0.000 | 8.83 | -64 -28 4 | L Middle Temporal Gyrus |
|  | 0.000 | 8.71 | -48 -20 -6 | L Middle Temporal Gyrus |
|  | 0.000 | 7.61 | -56 -4 -4 | L Superior Temporal Gyrus |
| 1824 | 0.000 | 7.34 | -4 4 70 | L SMA |
|  | 0.002 | 5.19 | 4 12 62 | R SMA |
|  | 0.002 | 5.06 | -2 16 50 | L SMA |
| 215 | 0.001 | 5.60 | -22 -6 -10 | L Amygdala |
|  | 0.009 | 4.02 | -32 2 -28 | L Amygdala |
| 283 | 0.003 | 4.74 | -20 4 8 | L Putamen |
|  | 0.030 | 3.21 | -10 -4 8 | L Thalamus |
|  | 0.032 | 3.17 | -20 -4 0 | L Pallidum |
| 136 | 0.005 | 4.44 | 0 -2 -4 | Third Ventricle* |
|  | 0.021 | 3.47 | 4 -4 -12 | WM* |
| 140 | 0.007 | 4.23 | -34 -60 -28 | L Cerebellum |
|  | 0.035 | 3.11 | -44 -68 -28 | L Cerebellum |
| 84 | 0.011 | 3.89 | -34 -50 36 | L Hippocampus |
| 9 | 0.013 | 3.79 | 22 -40 0 | R Hippocampus |
| 57 | 0.014 | 3.77 | 10 -8 -16 | R Thalamus |
| 58 | 0.014 | 3.76 | -16 -42 24 | L Posterior Cingulate Gyrus |
| 12 | 0.015 | 3.73 | 10 -36 -24 | R Cerebellum |
| 32 | 0.015 | 3.72 | -16 -26 -16 | L Parahippocampal Gyrus |
| 36 | 0.015 | 3.70 | -8 -18 54 | L SMA |
| 57 | 0.016 | 3.68 | 20 -42 22 | R Lateral Ventricle* |
|  | 0.028 | 3.27 | 12 -40 20 | R Retrosplenial Cortex |
| 32 | 0.016 | 3.66 | -4 -20 -24 | L Medulla |
| 35 | 0.020 | 3.50 | 18 -14 34 | WM* |
|  | 0.041 | 3.00 | 10 -16 28 | WM* |
| 114 | 0.021 | 3.49 | 34 -58 -32 | R Cerebellum |
|  | 0.037 | 3.06 | 30 -66 -24 | R Cerebellum |
| 38 | 0.021 | 3.47 | 54 -38 58 | R Superior Parietal Lobule |
| 34 | 0.023 | 3.42 | -6 -32 28 | WM* |
| 13 | 0.023 | 3.40 | 28 -26 16 | R Heschl's Gyrus |
| 7 | 0.024 | 3.39 | -18 -38 0 | L Hippocampus |
| 21 | 0.024 | 3.37 | 12 -14 58 | R SMA |
| 17 | 0.027 | 3.31 | -12 -10 32 | L Cingulate Gyrus |
| 14 | 0.027 | 3.29 | 62 -38 48 | R Inferior Parietal Lobule |
| 15 | 0.028 | 3.28 | 44 -40 -24 | R Fusiform Gyrus |
| 20 | 0.029 | 3.25 | 10 2 32 | R Cingulate Gyrus |
| 20 | 0.029 | 3.25 | -30 44 22 | L Middle Frontal Gyrus |
| 19 | 0.030 | 3.21 | 28 -28 -14 | R Parahippocampal Gyrus |
| 6 | 0.035 | 3.12 | 52 -32 58 | R Postcentral Gyrus |
| 7 | 0.035 | 3.12 | 38 36 18 | R Middle Frontal Gyrus |
| 7 | 0.036 | 3.10 | 20 -18 -22 | R Parahippocampal Gyrus |
| 17 | 0.036 | 3.09 | 42 -42 64 | R Postcentral Gyrus |
| 8 | 0.036 | 3.08 | 16 -32 14 | R Hippocampus |
| 6 | 0.040 | 3.02 | 54 38 10 | R Inferior Frontal Gyrus (p. triangularis) |
| 7 | 0.042 | 2.97 | -40 -22 34 | L Postcentral Gyrus |
| 7 | 0.044 | 2.94 | 4 -62 -16 | R Cerebellum |

* denotes regions in white matter or near ventricles.

Height threshold: T=2.83, p=0.006

Extent threshold: k=5 voxels, p=0.70

Expected false discovery rate: <=0.05

df: 1, 18

Smoothness (FWHM): 11.1 12.1 11.3mm = 5.6 6.0 5.7voxels

Search volume: 1523288cmm, 190411voxels, 927.9resels

Voxel size: 2 2 2mm (1 resel=190.06voxels)

Table S4: Correlation between age and select behavioral measures.

|  | **Age** | **PD score** | **C score** | **Perception Accuracy** | **Production Score** |
| --- | --- | --- | --- | --- | --- |
| **R(n=18):** | **1.000** | -0.193 | -0.012 | -0.238 | 0.218 |
|  |  | **1.000** | -0.601 | 0.444 | -0.133 |
|  |  |  | **1.000** | -0.358 | 0.229 |
|  |  |  |  | **1.000** | -0.135 |
|  |  |  |  |  | **1.000** |
|  |  |  |  |  |  |
| **R-sq(n=18):** | **1.000** | 0.037 | 0.000 | 0.057 | 0.048 |
|  |  | **1.000** | 0.362 | 0.197 | 0.018 |
|  |  |  | **1.000** | 0.128 | 0.053 |
|  |  |  |  | **1.000** | 0.018 |
|  |  |  |  |  | **1.000** |
|  |  |  |  |  |  |
| **p-value(n=18)** | **1.000** | 0.428 | 0.961 | 0.327 | 0.369 |
|  |  | **1.000** | 0.007* | 0.057 | 0.586 |
|  |  |  | **1.000** | 0.132 | 0.345 |
|  |  |  |  | **1.000** | 0.581 |
|  |  |  |  |  | **1.000** |

* denotes p<0.05.

Table S4: Correlation matrix of Age and select behavioral measures. PD score is the Personal Distress score of the IRI. C score is the Coldheartedness score of the PPI-R. Perception accuracy is the performance on the prosody perception task. Production score is the rating of the participants' performance on the prosody production task. Note that Age is not correlated with any of the behavioral measures described in the analyses (note the small R-square values), and was thus considered not to be a confounding factor in the correlation analyses described in the manuscript. Note that PD score and C score are anti-correlated, as expected.

**Table S5: Happy – Neutral prosody production contrast results.**

| **Cluster size (k)** | | **p(FDR-cor)** | | **Voxel T** | | **x,y,z {mm}** | | **Anatomical region** |
| --- | --- | --- | --- | --- | --- | --- | --- | --- |
| 226 | | 0.040 | | 5.91 | | -40 12 -34 | | L Medial Temporal Pole |
|  | | 0.040 | | 4.62 | | -46 8 -20 | | L Temporal Pole |
|  | | 0.040 | | 3.93 | | -30 6 -30 | | L Medial Temporal Cortex |
| 4163 | | 0.040 | | 5.72 | | -12 -60 -2 | | L Lingual Gyrus |
|  | | 0.040 | | 5.51 | | -8 -74 4 | | L Lingual Gyrus |
|  | | 0.040 | | 5.38 | | -20 -66 2 | | L Lingual Gyrus |
| 107 | | 0.040 | | 4.98 | | -34 20 22 | | L Inferior Frontal Gyrus* |
| 35 | | 0.040 | | 4.83 | | -4 -102 10 | | L Calcarine Gyrus |
| 80 | | 0.040 | | 4.67 | | 10 -12 -16 | | R Midbrain |
|  | | 0.040 | | 4.50 | | 4 -16 -22 | | R Midbrain |
| 48 | | 0.040 | | 4.52 | | 20 -34 -20 | | R Cerebellum |
| 46 | | 0.040 | | 4.48 | | -42 2 44 | | L Precentral Gyrus* |
| 33 | | 0.040 | | 4.22 | | 6 24 16 | | R Anterior Cingulate Cortex |
| 34 | | 0.040 | | 4.14 | | 44 2 -24 | | R Medial Temporal Cortex |
|  | | 0.040 | | 3.69 | | 46 10 -24 | | R Temporal Pole |
| 29 | | 0.040 | | 4.13 | | 18 -12 4 | | R Thalamus* |
| 31 | | 0.040 | | 4.12 | | -6 0 70 | | L SMA |
|  | | 0.040 | | 4.10 | | -10 2 62 | | L SMA |
| 62 | | 0.040 | | 4.10 | | 38 -68 22 | | R Middle Occipital Gyrus |
|  | | 0.040 | | 3.75 | | 34 -76 24 | | R Middle Occipital Gyrus |
| 12 | 0.040 | | 4.01 | | -8 28 32 | | L Middle Occipital Gyrus* | |
| 16 | 0.040 | | 4.00 | | 16 -16 20 | | R Caudate Nucleus* | |
| 9 | 0.040 | | 3.96 | | 40 16 -38 | | R Medial Temporal Pole | |
| 33 | 0.040 | | 3.96 | | -16 -40 10 | | Hippocampus | |
|  | 0.040 | | 3.95 | | -10 -34 8 | | Hippocampus | |
| 19 | 0.040 | | 3.95 | | -12 16 50 | | L Superior Frontal Gyrus | |
| 31 | 0.040 | | 3.92 | | 32 -8 22 | | R Posterior Insula | |
| 8 | 0.040 | | 3.89 | | -6 -22 12 | | L Thalamus | |
| 9 | 0.040 | | 3.86 | | -12 14 30 | | L Cingulate Cortex | |
| 7 | 0.040 | | 3.80 | | -10 54 28 | | L Superior Medial Gyrus | |
| 32 | 0.040 | | 3.80 | | 4 12 52 | | R SMA | |
| 12 | 0.040 | | 3.74 | | -10 22 62 | | L SMA | |
| 5 | 0.050 | | 3.63 | | -4 -32 -26 | | L Midbrain | |
| 8 | 0.050 | | 3.61 | | 10 -80 48 | | R Precuneus | |
| 6 | 0.050 | | 3.58 | | 24 -46 22 | | R Lateral Ventricle* | |

* = also active in perception (p<0.05 FDR; k>5; after SVC).

Height threshold: T=3.48, p=0.001

Extent threshold: k=5 voxels, p=0.64

Expected false discovery rate: <=0.05

df: 1, 18

Smoothness (FWHM): 12.6 12.9 12.6mm = 6.3 6.5 6.3voxels

Search volume: 1516016cmm, 189502voxels, 687.5resels

Voxel size: 2 2 2mm (1 resel=255.38voxels)

**Table S6: Question – Neutral prosody production contrast results.**

| **Cluster size (k)** | **p(FDR-cor)** | **Voxel T** | **x,y,z {mm}** | **Anatomical region** |
| --- | --- | --- | --- | --- |
| 2042 | 0.050 | 5.72 | 8 -70 34 | R Precuneus |
|  | 0.050 | 5.62 | -18 -68 2 | L Lingual Gyrus |
|  | 0.050 | 5.58 | -18 -50 2 | L Precuneus |
| 154 | 0.050 | 5.56 | -42 6 38 | L Precentral Gyrus* |
|  | 0.050 | 5.05 | -44 0 50 | L Precentral Gyrus |
| 39 | 0.050 | 5.10 | -10 26 34 | L Superior Medial Gyrus* |
| 35 | 0.050 | 4.70 | -14 4 66 | L SMA* |
| 19 | 0.050 | 4.64 | -32 -18 40 | Area 4p |
| 22 | 0.050 | 4.48 | 6 32 16 | R Anterior Cingulate Cortex |
|  | 0.050 | 3.91 | 2 40 16 | L Anterior Cingulate Cortex |
| 30 | 0.050 | 4.48 | -2 -48 66 | L Precuneus |
| 28 | 0.050 | 4.38 | 40 4 54 | R Middle Frontal Gyrus |
|  | 0.050 | 4.33 | 32 4 52 | R Middle Frontal Gyrus |
| 8 | 0.050 | 4.23 | 6 -56 -6 | R Cerebellum |
| 14 | 0.050 | 4.23 | 38 0 30 | R Inferior Frontal Gyrus |
| 5 | 0.050 | 4.21 | -22 -20 40 | WM |
| 23 | 0.050 | 4.19 | -32 20 24 | L Inferior Frontal Gyrus* |
|  | 0.050 | 4.03 | -34 20 16 | Area 44 |
| 44 | 0.050 | 4.15 | 6 16 58 | R SMA* |
| 6 | 0.050 | 4.14 | -36 48 26 | L Middle Frontal Gyrus |
| 16 | 0.050 | 4.08 | -14 -58 64 | L Precuneus |
| 5 | 0.050 | 4.06 | 28 20 -2 | R Putamen |
| 5 | 0.050 | 3.90 | -4 -70 60 | L Precuneus |

* = also active in perception (p<0.05 FDR; k>5; after SVC).

Height threshold: T=3.80, p=0.001

Extent threshold: k=5 voxels, p=0.56

Expected false discovery rate: <=0.05

df: 1, 18

Smoothness (FWHM): 12.2 12.4 12.2mm = 6.1 6.2 6.1voxels

Search volume: 1516016cmm, 189502voxels, 759.9resels

Voxel size: 2 2 2mm (1 resel=231.06voxels)

**Supporting Figures**

**Figure S1: Prosody perception and production performance.** Participants performed the prosody perception task with high accuracy, with a mean accuracy score of 0.91 (SD 0.11). The mean rating on the production task was 2.97 (SD 0.70). **Figure S2: Correlations between prosody perception performance and empathy measures.** Participants' performance on the prosody perception task was positively correlated with PD scores, a measure of affective empathy [r = 0.46; R-sq = 0.21; p(one-tailed) < 0.0287]. Prosody perception performance was negatively correlated with C scores, a measure thought to be associated with a lack of empathy [r = -0.47; R-sq = 0.22; p(one-tailed) < 0.0297]. **Figure S3: Regions involved in speech production and perception.** Regions involved in the production (red; p<0.05 FDR, T>2.14) and perception (green; p<0.05 FDR, T>2.83) of all speech conditions (all speech - rest). Regions common to both production and perception are shown in yellow.

**Figure S4: Regions involved in prosody production.** Regions involved in the production of “happy” (red; p<0.05; FDR; T>3.48), “sad” (blue; p<0.05; FDR; T>4.75), and “question” (green; p<0.05; FDR; T>3.80) prosody (compared against the “neutral” condition).

**Figure S5: Regions involved in prosody perception.** Regions involved in the perception of “happy” (red), “sad” (blue), and “question” (green) prosody (compared against the “neutral” condition). All effect size maps were thresholded at p<0.005 (uncorrected). No voxels in any of the three contrasts survive multiple comparisons correction at the whole-brain level.
