# Supplementary figures and images for: Common Premotor Regions for the Perception and Production of Prosody and Correlations with Empathy and Prosodic Ability
Source: PLoS One. 2010 Jan 20;5(1):e8759. doi: 10.1371/journal.pone.0008759 (PMC2808341; doi:10.1371/journal.pone.0008759)

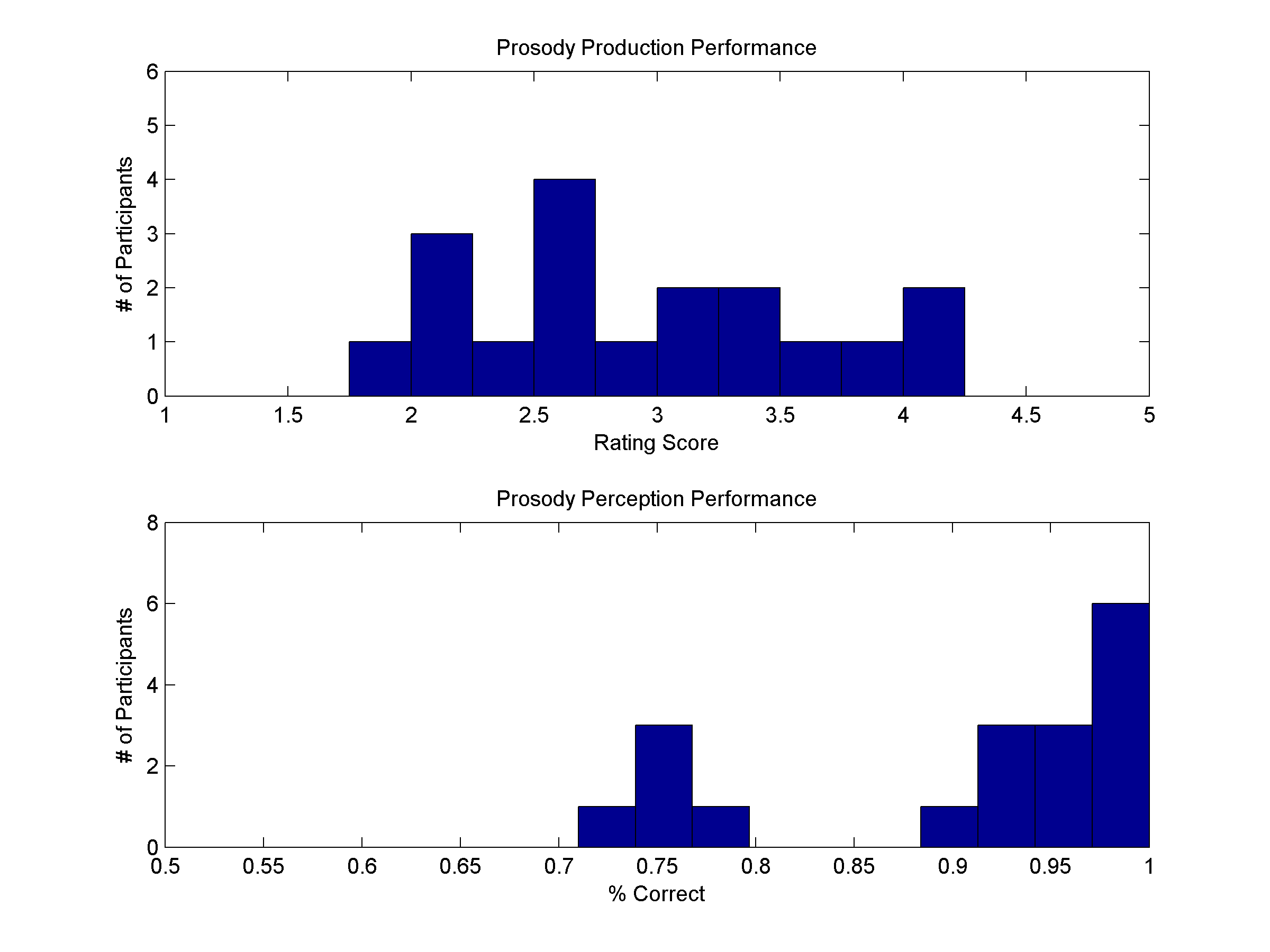

Supplement: Figure S1 — Prosody perception and production performance. Participants performed the prosody perception task with high accuracy, with a mean accuracy score of 0.91 (SD 0.11). The mean rating on the production task was 2.97 (SD 0.70). (1.30 MB TIF) [file pone.0008759.s002.tif]

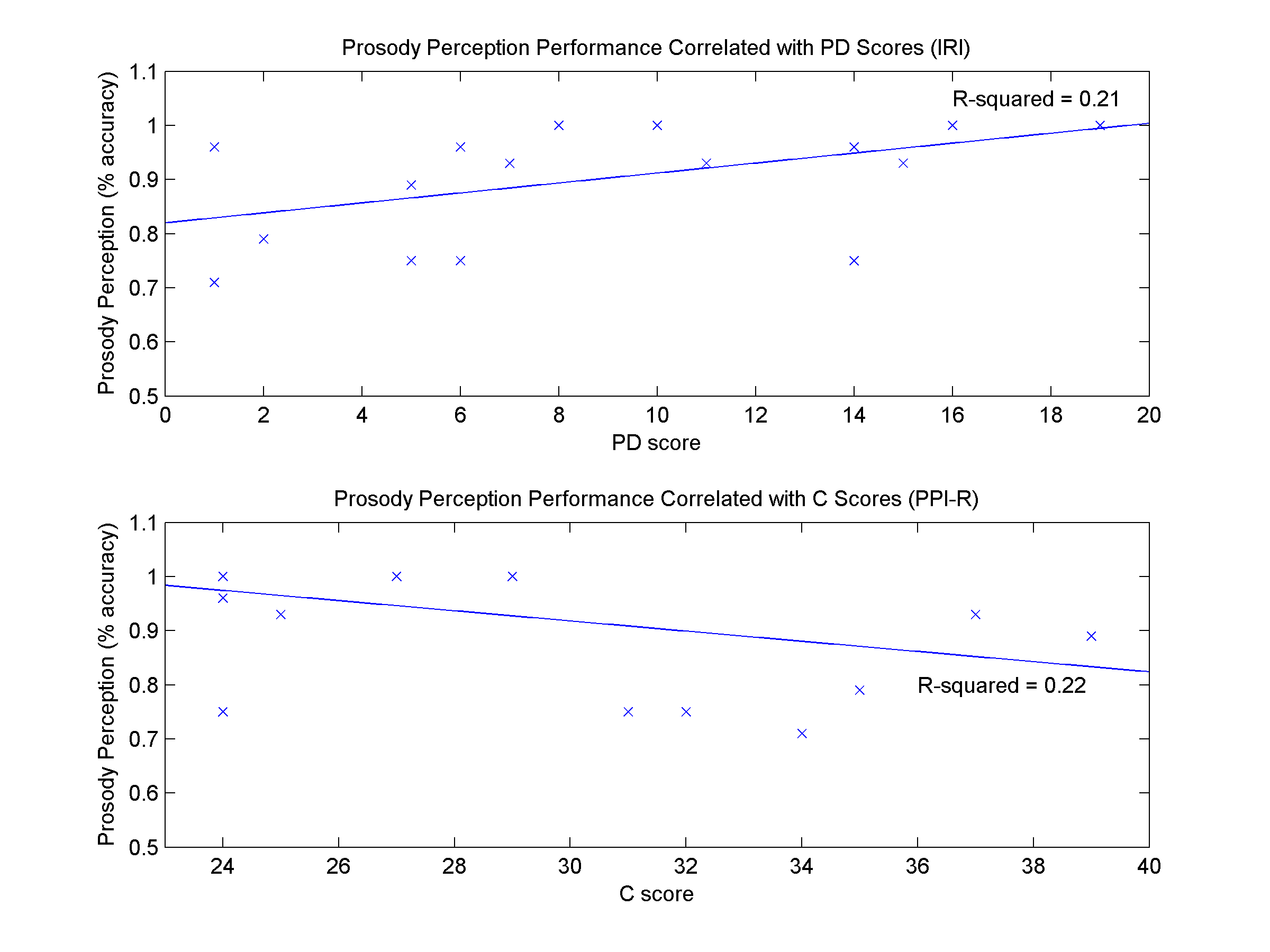

Supplement: Figure S2 — Correlations between prosody perception performance and empathy measures. Participants' performance on the prosody perception task was positively correlated with PD scores, a measure of affective empathy [r = 0.46; R-sq = 0.21; p(one-tailed) <0.0287]. Prosody perception performance was negatively correlated with C scores, a measure thought to be associated with a lack of empathy [r = −0.47; R-sq = 0.22; p(one-tailed) <0.0297]. (0.30 MB TIF) [file pone.0008759.s003.tif]

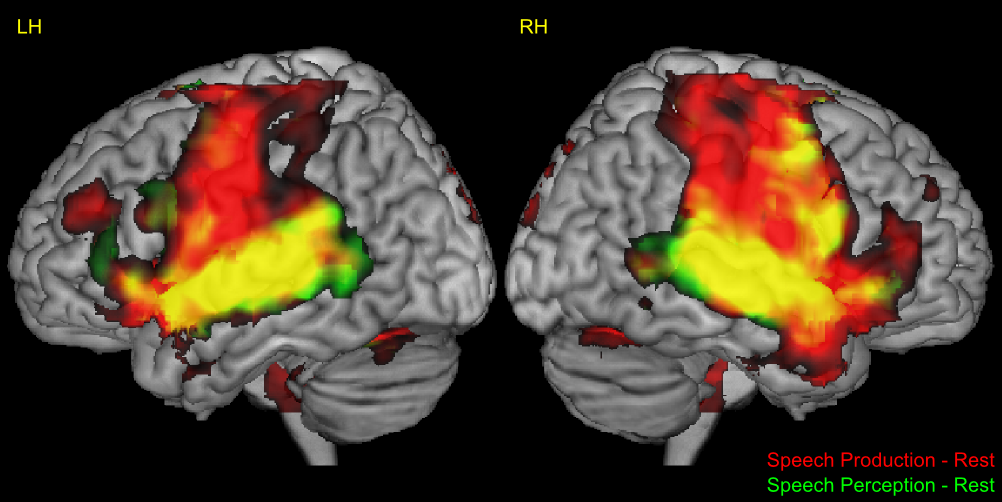

Supplement: Figure S3 — Regions involved in speech production and perception. Regions involved in the production (red; p<0.05 FDR, T>2.14) and perception (green; p<0.05 FDR, T>2.83) of all speech conditions (all speech - rest). Regions common to both production and perception are shown in yellow. (1.51 MB TIF) [file pone.0008759.s004.tif]

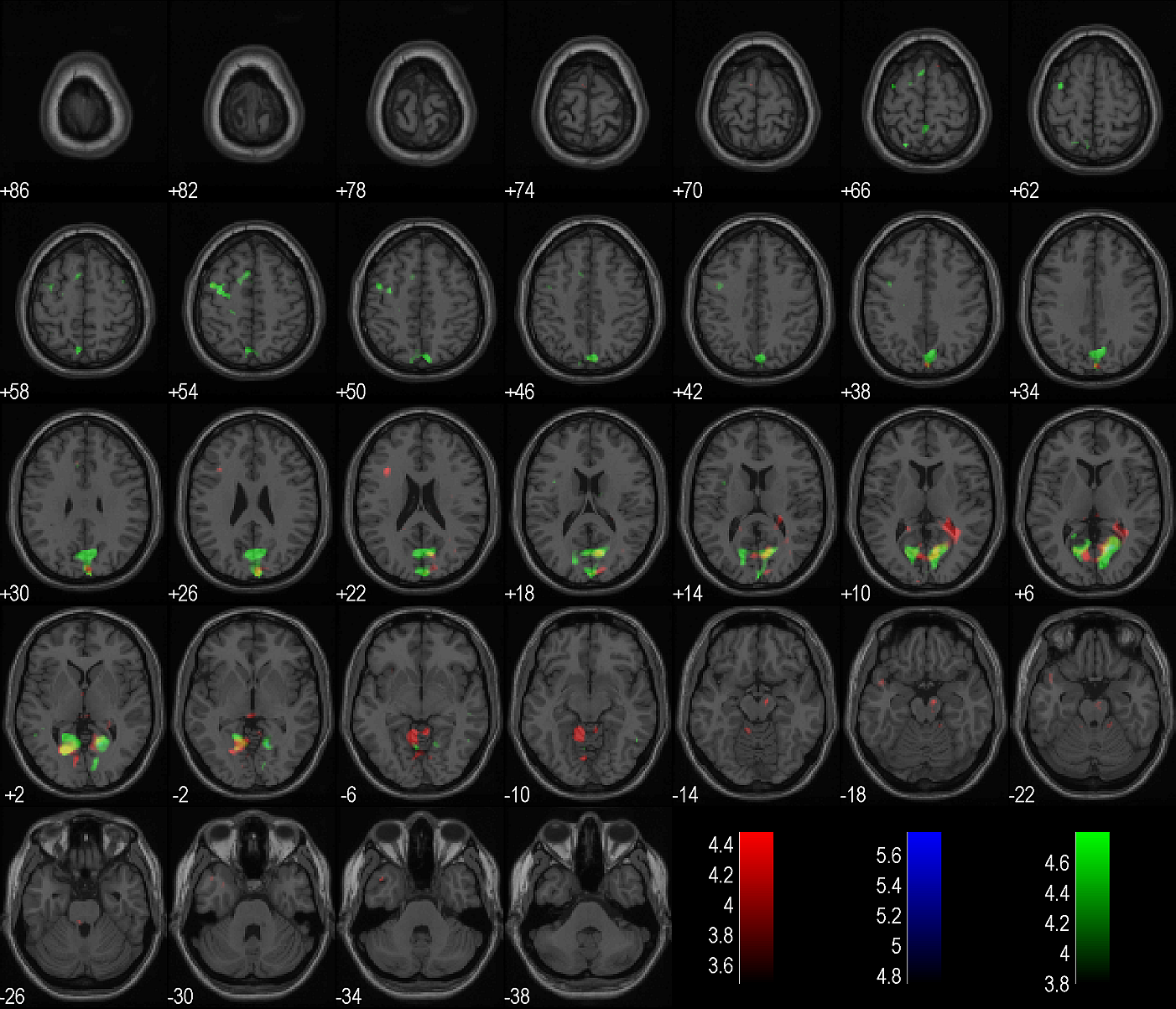

Supplement: Figure S4 — Regions involved in prosody production. Regions involved in the production of “happy” (red; p<0.05; FDR; T>3.48), “sad” (blue; p<0.05; FDR; T>4.75), and “question” (green; p<0.05; FDR; T>3.80) prosody (compared against the “neutral” condition. (5.07 MB TIF) [file pone.0008759.s005.tif]

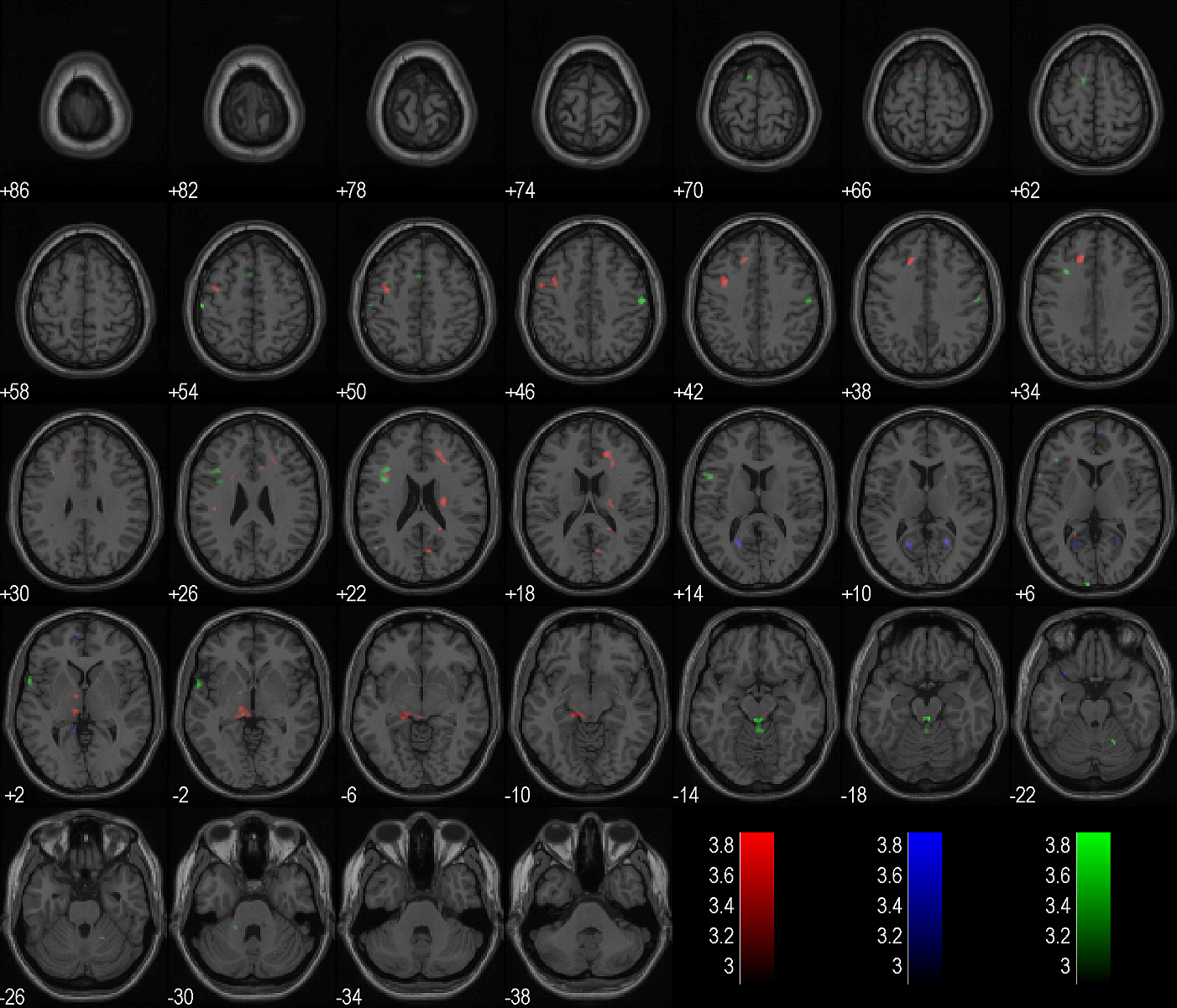

Supplement: Figure S5 — Regions involved in prosody perception. Regions involved in the perception of “happy” (red), “sad” (blue), and “question” (green) prosody (compared against the “neutral” condition). All effect size maps were thresholded at p<0.005 (uncorrected). No voxels in any of the three contrasts survive multiple comparisons correction at the whole-brain level. (5.06 MB TIF) [file pone.0008759.s006.tif]
